# The Endothelial Glycocalyx: Physiology and Pathology in Neonates, Infants and Children

**DOI:** 10.3389/fcell.2021.733557

**Published:** 2021-09-01

**Authors:** Alexandra Puchwein-Schwepcke, Orsolya Genzel-Boroviczény, Claudia Nussbaum

**Affiliations:** ^1^Division of Neonatology, Department of Pediatrics, Dr. von Hauner Children’s Hospital, University Hospital, LMU Munich, Munich, Germany; ^2^Department of Pediatric Neurology and Developmental Medicine, University of Basel Children’s Hospital, Basel, Switzerland

**Keywords:** glycocalyx, neonate, children, development, perfused boundary region, shedding

## Abstract

The endothelial glycocalyx (EG) as part of the endothelial surface layer (ESL) is an important regulator of vascular function and homeostasis, including permeability, vascular tone, leukocyte recruitment and coagulation. Located at the interface between the endothelium and the blood stream, this highly fragile structure is prone to many disruptive factors such as inflammation and oxidative stress. Shedding of the EG has been described in various acute and chronic diseases characterized by endothelial dysfunction and angiopathy, such as sepsis, trauma, diabetes and cardiovascular disease. Circulating EG components including syndecan-1, hyaluronan and heparan sulfate are being evaluated in animal and clinical studies as diagnostic and prognostic markers in several pathologies, and advances in microscopic techniques have enabled *in vivo* assessment of the EG. While research regarding the EG in adult physiology and pathology has greatly advanced throughout the last decades, our knowledge of the development of the glycocalyx and its involvement in pathological conditions in the pediatric population is limited. Current evidence suggests that the EG is present early during fetal development and plays a critical role in vessel formation and maturation. Like in adults, EG shedding has been demonstrated in acute inflammatory conditions in infants and children and chronic diseases with childhood-onset. However, the underlying mechanisms and their contribution to disease manifestation and progression still need to be established. In the future, the glycocalyx might serve as a marker to identify pediatric patients at risk for vascular sequelae and as a potential target for early interventions.

## Introduction

**FIGURE 1:**
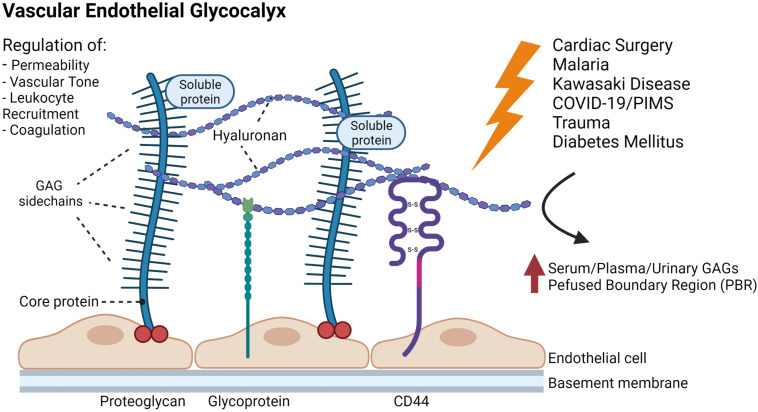
Schematic representation of the glycocalyx covering the endothelial cells of a blood vessel. The main membrane bound components of the EG include proteoglycans (e.g., syndecans and glypicans) with long glycosaminoglycan side-chains (GAGs) and glycoproteins (e.g., selectins and integrins). Hyaluronan, plasma proteins and soluble proteoglycans are integrated into the glycocalyx forming the so called endothelial surface layer. The EG is an important regulator of vascular function and homeostasis, and shedding of the EG has been suggested in different conditions and disease states in the pediatric population including cardiac surgery, trauma, infectious diseases and diabetes mellitus. Note: dimensions in the figure are not drawn to scale. The figure was created with Bio.Render.com.

The endothelial glycocalyx (EG), a complex and highly versatile brush-like carbohydrate-rich layer, lines the luminal endothelial surface of the whole vasculature including blood and lymphatic vessels. The structure and composition of the glycocalyx have been described in several excellent reviews and will not be covered in detail in this paper (Reitsma et al., 2007; Weinbaum et al., 2007; Couchman and Pataki, 2012). Briefly, the EG is mainly composed of proteoglycans, consisting of a core protein with attached long unbranched glycosaminoglycans (GAGs) and glycoproteins characterized by short, branched carbohydrate side chains. Together with associated plasma proteins, it forms the endothelial surface layer (ESL) (Figure 1). The core protein of proteoglycans is linked to the cell membrane (syndecans and glypicans) or secreted (e.g., versican, perlecan, agrin) (Couchman and Pataki, 2012). Among the bound GAGs, heparan sulfate is the most abundant, followed by chondroitin-/dermatansulfate, whereas hyaluronan, another structurally important GAG, is not firmly attached (Sarrazin et al., 2011). The composition and dimension of the EG varies within different types of blood and lymphatic vessels and ranges from approximately 0.3 to 0.5 μm in lymphatic collectors and blood capillaries to several micrometers in large arteries (Vink and Duling, 1996; Megens et al., 2007; Zolla et al., 2015).

Due to its central position within the vasculature, the EG is an important regulator of vessel function and homeostasis, including permeability, vascular tone, leukocyte recruitment and coagulation (Reitsma et al., 2007; Weinbaum et al., 2007). Similarly, in the lymphatic system the glycocalyx exerts an important role in limiting permeability and thereby contributes essentially to the drainage and transport of interstitial fluid and macromolecules (Zolla et al., 2015; Arokiasamy et al., 2019).

Since its first visualization more than 50 years ago by Luft (1966), the glycocalyx has gained increasing interest in cardiovascular research, especially throughout the last two decades. Numerous *in vitro* and *in vivo* studies have broadened our understanding of the EG’s function and its contribution to pathophysiological processes. For further information the reader is referred to recent comprehensive reviews on the topic (Pillinger and Kam, 2017; Cosgun et al., 2020). However, almost all studies have been performed in adults, leaving a knowledge gap concerning the composition and function of the EG in the developing organism. This review aims to summarize our current understanding of the EG in the fetus, neonate and in children and its involvement in pathological processes in the pediatric population, thereby identifying open questions for future research.

## Assessment of the Endothelial Glycocalyx in Pediatric Clinical Studies

While the scientific interest in the EG has significantly increased throughout the last decades, the assessment of the EG *in vivo* remains challenging. Using conventional intravital microscopy, Vink and Duling were the first to indirectly visualize the EG in vessels of the mouse cremaster muscle by demonstrating an exclusion zone near the vessel wall for flowing erythrocytes or a fluorescent plasma marker unable to penetrate the EG (Vink and Duling, 1996). Advanced imaging techniques such as multiphoton laser scanning microscopy may offer the potential to directly image the glycocalyx *in vivo* in animal models using fluorescent dyes or antibodies targeting EG components (Wu et al., 2017). However, none of these approaches is currently practicable for *in vivo* assessment of the EG in humans. Nieuwdorp et al. (2006b) applied a tracer dilution method to gain estimates of systemic glycocalyx volume in adult test persons, but the validity of this technique has been questioned (Michel and Curry, 2009) and ethical concerns hamper its application in the pediatric population. Currently, evaluation of the EG in clinical studies is mainly based on two principles: (i) measurement of circulating glycocalyx components such as syndecan-1, hyaluronan, heparan sulfate and chondroitin sulfate in the plasma/serum and urine as an indicator for glycocalyx shedding and (ii) videomicroscopic assessment of the EG in vessels of the microcirculation (Cerny et al., 2017). Both methods only provide indirect information on the EG, and in the pediatric population, specific challenges need to be overcome.

While measurements of circulating EG biomarkers are relatively easy to realize in adults, obtaining the necessary blood samples in children for research purposes alone is disputable. Furthermore, especially in preterm newborns, drawing the sample volumes required for accurate analyses is critical due to the low total blood volume. Newer videomicroscopy techniques, including Orthogonal Polarization Spectral (OPS), Sidestream Dark Field (SDF), and Incident Dark Field (IDF) imaging, have enabled *in vivo* visualization of the human microcirculation, including neonates and children (Genzel-Boroviczeny et al., 2002; Erdem et al., 2019). These video sequences can be used to measure the local microvascular EG based on changes in vessel diameter. One of the best established and validated parameters is the so-called perfused boundary region (PBR), resembling the luminal part of the EG partially accessible to flowing erythrocytes (Lee et al., 2014; Eickhoff et al., 2020). Changes in glycocalyx composition or shedding of the EG, allowing erythrocytes to further penetrate into the EG, are reflected by an increase in PBR. The PBR has been evaluated in various clinical studies in adults and correlated to patient outcome (Vlahu et al., 2012; Dekker et al., 2019; Rovas et al., 2019; Beurskens et al., 2020). Performing videomicroscopic studies in children and especially in infants and neonates is challenging due to the need for minimal movement during image acquisition.

## Physiological Properties of the EG in the Fetus and Neonate

### Role of the EG in Blood Vessel Formation

In the developing embryo, blood vessel formation and growth are necessary at an early stage to guarantee cellular supply with oxygen and nutrients. In general, two distinct processes can be distinguished in the development of vasculature. Vasculogenesis describes the *de novo* formation of vessels by differentiation, proliferation and migration of endothelial progenitor cells. In contrast, angiogenesis characterizes the generation of new vessels from existing ones by sprouting and intussusception (i.e., splitting of an existing vessel) (Conway et al., 2001; Naito et al., 2020). Components of the glycocalyx have been shown to be critically involved in both processes (Iozzo and San Antonio, 2001; Piecewicz and Sengupta, 2011). It is long known that pro-angiogenic factors critical for vasculo- and angiogenesis, including vascular endothelial growth factor (VEGF) and fibroblast growth fact-2 (FGF-2), bind to heparan sulfate proteoglycans (HSPG), the most abundant component of the EG (Yayon et al., 1991; Gitay-Goren et al., 1992; Lundin et al., 2000). As demonstrated by Harfouche et al. (2009), differentiation of embryonic stem cells into endothelial cells is paralleled by an increase in the synthesis of di- and trisulfated heparan sulfate glycosaminoglycans (HSGAG). Vice versa, inhibition of HSGAG sulfation by treatment with sodium chloride or digestion of HSGAGs by heparinase led to a significantly lower expression of endothelial markers such as von Willebrand factor and angiopoetin-2 (Harfouche et al., 2009). These *in vitro* findings were validated in zebrafish embryos showing that knock-down of the enzyme N-deacetylase/N-sulfotransferase 1 (NDST1), which is critical for posttranslational sulfation of glycosaminoglycans, led to impaired vessel formation (Harfouche et al., 2009).

Syndecan-2, a plasma membrane-bound HSPG expressed on human microvascular endothelial cells (EC), is upregulated under stimulation with FGF or VEGF. Inhibition of Syndecan-2 gene transcription using antisense oligonucleotides led to impaired EC adhesion (i.e., attachment of EC to fibronectin coated culture dishes), spreading (i.e., number of attached ECs showing extended cytoplasm) and capillary tube formation *in vitro* (Noguer et al., 2009). *In vivo*, knock-down of Syndecan-2 by injection of morpholino designed against the 5′ UTR region of Syndecan-2 mRNA, led to impaired VEGF-dependent angiogenic sprouting in the zebrafish (Chen et al., 2004). These studies point at the importance of the EG, and HSPGs in particular, during vasculogenesis and angiogenesis. As reviewed by Iozzo and San Antonio, HSPGs act in concert with pro-angiogenic factors to control vascular development by providing a depot for these factors, limiting their diffusion and promoting receptor-ligand interaction and intracellular signaling (Iozzo and San Antonio, 2001).

More recent studies by the group of D’amore investigated the function of endomucin (EMCN), an integral sialoglycoprotein present in the EG of capillaries and veins, during angiogenesis (Park-Windhol et al., 2017; LeBlanc et al., 2019). Using a model of mouse retinal vascularization, it was demonstrated that silencing of EMCN resulted in a significant reduction of retinal vessel density and branching (Park-Windhol et al., 2017). Further analyses with human retinal endothelial cells lacking or overexpressing EMCN corroborated the role of EMCN in VEGF-induced signaling pathways by modulating internalization of the VEGF receptor 2 (VEGFR2), thereby regulating EC proliferation and migration (LeBlanc et al., 2019). It was recently shown that this effect of EMCN was dependent on N-glycosylation of its extracellular domain (Hu et al., 2020). Taken together, these studies highlight the essential involvement and contribution of the EG in vessel formation.

### Characterization of the EG in the Fetus and Neonate

One of the challenges in interpreting the results of studies on the EG in pediatric diseases is the lack of reference values. Recent investigations in animals and humans have provided evidence that aging is accompanied by a reduction in the EG size possibly due to increased EG shedding in combination with decreased synthesis of glycocalyx components (Machin et al., 2018; Majerczak et al., 2019). The glycocalyx thickness in sublingual capillaries of old study participants (mean age 60 ± 2 years) compared to young (mean age 29 ± 1 years) decreased by around 30%. Interestingly, a significant reduction in glycocalyx thickness was also demonstrated in the aging lymphatic vasculature of the rat mesentery with a decrease by more than 50% in 24-month old animals versus 9 month old animals (note: 1 month of life in a rat equals about 3 years in a human) (Zolla et al., 2015). Considering the profound physiological changes occurring in the growing fetus, neonate and child, it seems very likely that the EG is also subject to age-dependent variations. At present, very limited data is available on the ontogeny of the EG. As reported by Henderson-Toth et al. (2012), the EG can be detected in the dorsal aorta of quail embryos at an early developmental stage (14 somites) as soon as blood flow commences. Immunohistochemistry confirmed the presence of functionally important EG components, including hyaluronic acid, heparan sulfate and chondroitin sulfate. Selective enzymatic digestion of these components demonstrated a role of hyaluronan (and chondroitin-/dermatan-sulfate) in maintaining blood flow as well as vascular barrier function, thereby emphasizing the functional importance of the EG at this early developmental stage. Using SDF-imaging for PBR measurements in the cutaneous microcirculation, we have recently shown that the endothelial glycocalyx in preterm and term neonates depends on gestational age at birth (Puchwein-Schwepcke et al., 2021). Intriguingly, we observed an inverse correlation of the EG dimension with gestational age, i.e., the most immature neonates exhibited the thickest EG (represented by low PBR values). Whether this finding reflects the functionally importance of the EG in vascular development remains speculative due to the observational nature of the study. Longitudinal follow-up in the group of preterm infants further demonstrated an effect of postnatal age on the EG with a gradual decrease of EG thickness (increase in PBR). This effect was most pronounced in the group of extremely preterm neonates resulting in significantly higher PBR values (smaller EG) when reaching term age compared to term born neonates. This acceleration of PBR changes over time might be due to the frequent presence of multiple EG stressors (e.g., hyperglycemia, sepsis, reactive oxygen species) and could possibly contribute to a higher vascular vulnerability in this patient group.

Interestingly, PBR values reported for neonates and infants are consistently higher than in adults. In healthy mature newborns (mean age 3 days) the PBR was 2.14 ± 0.25 μm (Puchwein-Schwepcke et al., 2021) versus a PBR of 1.88 ± 0.2 μm measured in healthy adults (mean age 20.7 years) (Astapenko et al., 2019). Likewise, infants with cardiac defects (mean age 8.9 month) had a higher baseline PBR than adult cardiac patients (median age 64–69 years) before undergoing surgery on cardiopulmonary bypass (2.5 μm [2.44–2.7 IQR] vs. 2.0 ± 0.2 μm, respectively) (Nussbaum et al., 2015; Dekker et al., 2019; Dekker et al., 2020). At present it remains unclear whether these differences in PBR magnitude are due to methodological differences (e.g., measurements obtained sublingually versus the fossa auricularis of the ear conch) or truly reflect an age-dependence in PBR values.

Further studies in various age groups and in larger cohorts are needed to better understand the natural course of EG development and establish normal values necessary for the implementation of EG measurements in the clinical routine.

## Pathology of the EG in Acute Childhood Diseases and Chronic Conditions With Childhood-Onset

Shedding of the glycocalyx has been observed in many acute and chronic diseases in adults characterized by inflammation, endothelial dysfunction and microangiopathy, indicating its crucial role in the homeostasis of the microvasculature. In addition, acute events such as surgery or trauma have been shown to affect the glycocalyx, and patient outcome seems to be directly related to the extent of glycocalyx damage (Ostrowski and Johansson, 2012; Qi et al., 2021). In the pediatric population, information on disease-related EG alterations is still limited. Most data stems from studies evaluating the glycocalyx after pediatric heart surgery or pediatric trauma. Table 1 lists the clinical trials investigating the EG with respect to different pathologies in neonates, infants and children.

**TABLE 1:** Clinical studies investigating the EG in the pediatric population.

	Study type	EG parameters	*n*	Mean age	Major findings
**Pediatric heart surgery**					
Nussbaum et al. (2015)	Longitudinal cohort study	PBR (SDF imaging)	40 patients (36 with CPB, 4 without CPB)	CPB group: 8.9 months [0.2–29] w/o CPB: 9 months [0.2–31]	Increase in PBR after surgery on CPB
Bruegger et al. (2015)	Prospective cohort study	Serum syndecan-1, HA	42	7 months (2.9–23)	Increase of circulating HA and syndecan-1 associated with the ischemic impact
Pesonen et al. (2016)	2 double blinded placebo-controlled trials	Plasma syndecan-1	40 (1st trial), 45 (2nd trial)	1st trial: 7 days (1–27), 2nd trial: 0.37 years (0.15–1.36)	Lower syndecan-1 plasma levels after high-dose steroid treatment in complex heart surgery
de Melo Bezerra Cavalcante et al. (2016)	Prospective cohort study	Plasma syndecan-1	289	3.0 years (SD: ± 4.4)	Association of higher syndecan-1 levels with poorer outcomes and postoperative acute kidney disease
Ferrer et al. (2018)	Prospective cohort study	urinary syndecan-1	86	< 2.0 years: 61.2%	Higher postoperative urine syndecan-1 levels in patients with acute kidney injury
Bangalore et al. (2021)	Prospective cohort study	Plasma HS	27	4.9 months (1–22 months)	Association of circulating HS with metabolic acidosis, renal dysfunction and capillary leak after CPB
**Pediatric trauma**					
Richter et al. (2019)	Prospective cohort study	Plasma syndecan-1, angiopoetin-1 and angiopoetin-2	64 (52 trauma, 12 controls)	Trauma: 9.7 years (6.2–13.6), controls: 5 years (1.8–15)	Higher angiopoetin-2 levels associated with worse clinical outcome, pos. correlation of syndecan-1 and angiopoetin-2
Russell et al. (2018)	Prospective cohort study	Plasma syndecan-1 and hcDNA	211 (149 trauma, 62 controls)	Trauma: 8.3 years (4.6–12.3), controls 6.24 ±6.2 years	Highest syndecan 1 levels correspond to highest hcDNA levels and poor outcome
**Pediatric inflammatory and infectious diseases**					
*Kawasaki disease (KD)*					
Ohnishi et al. (2019)	Prospective cohort study	Plasma syndecan-1, HA	103 (70 complete KD, 18 febrile controls, 15 afebrile controls)	CAL (coronary artery lesions): 27 months (3–121), CAL negative: 18.5 (1–88)	Higher syndecan-1 and HA levels in KD compared to febrile and afebrile controls
Luo et al. (2019)	Prospective cohort study	Plasma syndecan-1	203 (119 KD, 43 healthy children, 40 children with febrile disease)	26 months (16.0–43.75)	Higher syndecan-1 levels in KD compared to matched febrile and afebrile controls
*COVID-19/PIMS*					
Fraser et al. (2021)	Case report	Plasma HA	1 pt., 20 controls	15 years [IQR 8]	Increased HA in a patient suffering from PIMS compared to controls
*Malaria*					
Yeo et al. (2019a)	Retrospective analysis of frozen samples of a prospective cohort study	Urinary GAGs	85	Uncomplicated Malaria: 3.1 years (0.5–7.8), complicated malaria: 3.6 years (0.6–7.2)	Higher urine excretion of GAGs in malaria groups compared to healthy children
Lyimo et al. (2020)	Cross-sectional study	PBR (IDF imaging), plasma sulfated GAGs	119 (healthy: 31, non-malaria fever NMF: 7, uncomplicated malaria UM: 12, severe malaria SM: 69)	Healthy: 2.5 years (0.8–4.3), NMF: 2.28 years (1.0–4.), UM: 5.5 years (1.1–10.1), SM: 4.1 years (0.6–10.0)	Increased PBR in patients with SM; sulfated GAGs higher in patients with complicated malaria compared to UM; positive association between HA and PBR
**Diabetes mellitus**					
Nussbaum et al. (2014)	Observational study	Glycocalyx thickness (SDF imaging)	14 patients, 14 controls	patients: 13.6 [9.9–14.4], controls: 11.6 [9.7–14]	Reduced EG thickness in diabetic children compared to controls; inverse correlation of EG with blood glucose levels

### EG in Pediatric Heart Surgery

Surgery on cardiopulmonary bypass has been shown to acutely and severely affect the integrity of the EG in adults (Rehm et al., 2007). In children undergoing cardiac surgery on cardiopulmonary bypass (CPB), an increase of circulating hyaluronan and syndecan-1 was witnessed in dependence of the ischemic impact indicating acute shedding of the EG (Bruegger et al., 2015). This was further confirmed in a longitudinal cohort study investigating 40 children that underwent cardiac surgery (36 with and four without CPB) using SDF-imaging to visualize the microcirculation at the ear conch (Nussbaum et al., 2015). A significant reduction in glycocalyx thickness (indicated by an increased PBR) was observed after cardiac surgery with cardiopulmonary bypass compared to preoperative values. In contrast, no significant change in PBR was observed in control patients subjected to a different procedure requiring general anesthesia (cleft palate surgery, cardiac catheterization), indicating a direct effect of the cardiopulmonary bypass in perturbation of the microvascular glycocalyx in pediatric heart surgery (Nussbaum et al., 2015). Similar results have been obtained in adult patients undergoing coronary bypass operation on CPB, demonstrating a significant increase in PBR during surgery. However, the time course of PBR changes described in adults differs from that in infants. While PBR values were shown to further increase during the first three postoperative days in adults following surgery on cardiopulmonary bypass (Dekker et al., 2019, 2020), in infants PBR values were already decreasing 24 h after surgery (Nussbaum et al., 2015). As the studies vary largely with respect to the underlying cardiac disease (congenital heart defect vs. coronary artery disease), surgical procedures applied and presence of cardiac risk factors, it is impossible to draw a conclusion from these studies regarding possible age-dependent differences in shedding and recovery of the glycocalyx.

In two double-blinded, randomized, placebo-controlled trials, syndecan-1 plasma levels were evaluated in neonates subjected to open heart surgery (neonatal trial) and in infants undergoing correction of a ventricular septal defect (VSD trial) to determine whether high-dose steroid treatment might have a protective effect on the glycocalyx. The authors could prove that in complex heart surgery in neonates, high-dose steroid treatment resulted in lower syndecan-1 levels compared to a placebo group. However, there were no differences in syndecan-1 levels between treatment and placebo groups in older children after VSD repair (Pesonen et al., 2016).

In a prospective cohort study on 289 children undergoing cardiac surgery, higher syndecan-1 levels were associated with poor outcomes and postoperative acute kidney disease (de Melo Bezerra Cavalcante et al., 2016). Similar results were found in a prospective cohort study on 86 pediatric patients recovering from heart surgery. Postoperative urinary syndecan-1 was collected within 2 h after surgery and was higher in patients suffering from acute kidney injury in the follow-up. In addition, the prediction of acute kidney injury in a risk-stratified statistical model of clinical outcome was improved after adding urinary syndecan-1 (Ferrer et al., 2018). These data were recently confirmed and expanded by Bangalore et al. (2021), demonstrating an association of the amount of circulating heparan sulfate with metabolic acidosis, renal dysfunction and capillary leak in 27 neonates and infants following cardiopulmonary bypass surgery.

Collectively, these studies provide univocal evidence for EG alterations in pediatric cardiac surgery contributing to adverse outcomes. Thus, assessment of the EG might offer the potential to identify patients at risk for postoperative complications and serve as a monitoring parameter to evaluate treatment strategies aiming at EG restoration.

### EG in Pediatric Trauma

Multiorgan failure after pediatric trauma has been discussed to be associated with an imbalanced inflammatory reaction that may lead to endothelial disruption and impairment of the glycocalyx. An increase of endothelial-derived angiopoietins (angiopoietin-1 and angiopoietin-2) indicates a developing endotheliopathy, whereas circulating syndecan-1 can be interpreted as a sign of glycocalyx injury. In a prospective cohort study, 52 pediatric trauma patients were compared to 12 pediatric controls with respect to angiopoietin levels, syndecan-1 levels and clinical outcome. The authors could show that higher angiopoietin-2 levels were associated with worse clinical outcomes and were positively correlated to syndecan-1 levels. This may indicate that glycocalyx injury results in adverse outcome (Richter et al., 2019).

Similar findings were observed in another prospective cohort study on 149 pediatric trauma patients and 62 pediatric controls studying the role of histonic DNA (hcDNA) as a marker of damage-associated molecular patterns (DAMPs) and circulating syndecan-1 levels as a marker of EG shedding (Russell et al., 2018). Syndecan-1 levels were evaluated in relation to hcDNA levels at admission and after 24 h. Control patients had low levels of both syndecan-1 and hcDNA, whereas these parameters were significantly higher in the pediatric trauma group, with the highest hcDNA levels corresponding to the highest levels of syndecan-1 and poor outcome. This indicates a link between trauma-induced extracellular hcDNA release and endothelial glycocalyx degradation. However, the causality of the association and the underlying mechanisms still need to be established.

### EG in Pediatric Inflammatory and Infectious Disease

Similarly, infectious diseases may result in acute effects on the microvasculature and the glycocalyx. During sepsis, shedding of the endothelial glycocalyx has been well established in the adult population and linked to mortality (Puskarich et al., 2016; Rovas et al., 2019; Beurskens et al., 2020; Saoraya et al., 2021). In the pediatric population, primarily Kawasaki disease and Malaria were studied for their association with glycocalyx damage.

#### Kawasaki Disease (KD)

Serum syndecan-1 and hyaluronic levels were analyzed in a prospective cohort study of 70 children with KD, 18 febrile controls and 15 afebrile controls. Patients suffering from KD had higher serum levels of syndecan-1 and hyaluronan, indicating EG damage. Moreover, patients that developed coronary artery lesions in the follow-up had higher levels of these parameters in the blood than those who didn’t, with serum hyaluronan being a highly contributive predictor of coronary involvement (Ohnishi et al., 2019).

Similar results were obtained in a prospective cohort of 120 pediatric patients with acute KD that were compared to a group of 43 matched healthy and 40 matched febrile controls. Patients suffering from KD had significantly higher levels of syndecan-1 in the plasma compared to febrile and healthy controls. Moreover, syndecan-1 levels were higher in patients suffering from coronary artery involvement than in uncomplicated Kawasaki disease (Luo et al., 2019).

#### COVID-19

During the COVID-19 pandemic, a novel syndrome termed PIMS (pediatric inflammatory, multisystem syndrome) or MIS-C (multisystem inflammatory syndrome in children) has emerged in the pediatric population following infection with SARS-CoV-2 (Feldstein et al., 2020; Whittaker et al., 2020). This severe hyperinflammatory condition shares similarities with Kawasaki disease and as with KD, increased levels of glycocalyx degrading enzymes (MMP-7) and hyaluronan have been reported, suggesting shedding of the endothelial glycocalyx (Fraser et al., 2021). Interestingly, in an experimental cell model (human H1299 cells, derived from type 2 alveolar cells) it was shown that SARS-CoV-2 requires cell surface heparan sulfate to promote binding and infection of host cells via angiotensin-converting enzyme (Clausen et al., 2020). Data from post-mortem studies in adults with severe courses of COVID-19 revealed direct involvement of the endothelial cells with widespread endothelitis (Varga et al., 2020). Furthermore, shedding of syndecan-1 and heparan sulfate and an increase in the PBR (i.e., decreased glycocalyx thickness) have been demonstrated during acute COVID-19 disease in adult patients (Stahl et al., 2020; Fernández et al., 2021; Rovas et al., 2021). While PIMS is also characterized by multiorgan involvement, it typically occurs weeks after the initial infection with SARS-CoV-2, which itself may have presented only with mild symptoms or even asymptomatic. Therefore, it is currently unclear whether the supposed disturbance of the glycocalyx in PIMS results from a direct effect of the virus on the endothelium or is rather a consequence of systemic inflammation. As PIMS is a relatively rare condition with reported incidence rates of 2 in 100,000 (Dufort et al., 2020), systematic research on its pathogenesis and the role of the glycocalyx remains a challenge.

#### Malaria

Urinary GAGs can be used as a marker of glycocalyx damage. Frozen urine samples of a prospective cohort study from 1994–1995 were thawed and analyzed for glycocalyx damage in three groups- healthy controls (10 children), children with uncomplicated malaria (20 children) and children suffering from cerebral malaria (55 children). Total urine excretion of GAGs was higher in pediatric malaria patients (mean age ∼ 4 years) compared to healthy children and inversely related to plasma nitrate and nitrite levels; however, no difference was seen between infants with cerebral malaria compared to those with uncomplicated disease. The authors concluded that this was a sign of glycocalyx breakdown leading to impaired endothelial nitric oxide (NO) production (Yeo et al., 2019a). By contrast, a study in adult malaria patients from the same group demonstrated significant differences in urinary GAGs between severely affected malaria patients (mean age 25 years) versus patients with a moderate course of disease (mean age 27 years) (Yeo et al., 2019b). The authors hypothesized that these differences might be due to more generalized vascular activation and dysfunction in adult malaria patients compared to children, where vascular dysfunction may possibly be limited to the cerebral microcirculation. In addition, it is conceivable that age dependent differences in glycocalyx breakdown and urinary elimination of GAGs might contribute to the observed differences (Sabir et al., 2020).

In a cross-sectional study on glycocalyx loss in pediatric malaria patients, authors assessed glycocalyx thickness *in vivo* by incident dark Field-imaging and glycocalyx degradation parameters in the plasma. As such, the PBR was increased in severe malaria patients indicating a loss of glycocalyx. Similarly, sulfated GAGs in the plasma were significantly higher in patients with severe malaria compared to those with uncomplicated malaria. There was a positive association between hyaluronic acid and PBR, suggesting that the loss of glycocalyx is related to disease severity (Lyimo et al., 2020).

#### EG in Children and Adolescents With Diabetes Mellitus

Many chronic diseases with long-term vascular sequelae are well known to affect the glycocalyx and the microvasculature. Diabetes mellitus type 1 and 2 belong to best-studied conditions with respect to the impact of experimental hyperglycemia on the endothelial glycocalyx (Zuurbier et al., 2005; Nieuwdorp et al., 2006b) as well as changes of the microcirculation and the EG in adult diabetic patients (Nieuwdorp et al., 2006a; Broekhuizen et al., 2010; Dogné et al., 2018; Wadowski et al., 2020). By contrast, only limited data is available on the effect of diabetes mellitus on the EG in the pediatric population. Indirect evidence for a possible impairment of the EG in children with diabetes mellitus stems from studies investigating the hyperemic response to a heat stimulus (Shore et al., 1991; Shah et al., 2015) or following arterial occlusion (Järvisalo et al., 2004; Pillay et al., 2018; Cao et al., 2021), consistently demonstrating endothelial dysfunction with impaired flow-mediated dilation. As the EG was shown to function as a mechanosensor regulating vascular tone in response to increased shear stress (Florian et al., 2003; Curry and Adamson, 2012; Dragovich et al., 2016), the finding of endothelial dysfunction in diabetic children is suggestive of EG alterations in these patients. This notion was supported by an observational study of 14 children between 9 and 14 years of age with diabetes type 1, demonstrating reduced glycocalyx thickness in video recordings of the sublingual microcirculation compared to a control group of 14 children. Furthermore, a significant inverse correlation between serum glucose levels and glycocalyx thickness was observed, suggesting a direct harmful effect of blood sugar levels on the glycocalyx (Nussbaum et al., 2014).

## Outlook and Future Areas of Research

In view of the importance of the EG for vascular integrity and the possible deleterious effects of EG destruction in acute and chronic diseases, methods to quickly assess the EG’s condition in patients would be of high relevance for the clinician. Especially in intensive care medicine, bedside approaches yielding fast results could help to identify patients at risk for adverse outcome and guide clinical decision making. As shown in the GlycoNurse study, after theoretical and practical training, nurses were able to perform high quality PBR measurements in patients of the emergency department and the intensive care unit in less than 10 min duration using a handheld videomicroscope and automated analysis software (Rovas et al., 2018). PBR values showed a high level of inter- and intraobserver reliability and an association with clinical markers of disease severity including mean arterial blood pressure, C-reactive protein levels as a marker of inflammation and SOFA score as an assessment tool for organ failure. Despite these promising results, before EG measurements can be implemented into clinical routine, further studies on larger patient numbers are needed to establish normal values in different age groups, define cut-off values for certain disease entities and evaluate the diagnostic and prognostic usefulness in predicting patient outcome.

In the last decade, the EG has evolved as a possible target for novel treatment strategies aiming at protection or reconstitution of the EG (Becker et al., 2010). Therapeutic approaches evaluated *in vitro* and *in vivo* include reduction of glycocalyx degradation by attenuating inflammation, e.g., by administration of corticosteroids (Chappell et al., 2009a; Pesonen et al., 2016; Brettner et al., 2019) and inhibition of EG degrading enzymes such as heparinase and metallo-matrix proteinases (Chappell et al., 2009b; Mulivor and Lipowsky, 2009; Zeng et al., 2014; Mensah et al., 2017). Furthermore, administration of glycocalyx and plasma components (e.g., sulodexid and albumin) and colloids (e.g., 6% Hydroxyethyl starch) have shown potential benefit in restoring the EG (Broekhuizen et al., 2010; Margraf et al., 2018; Aldecoa et al., 2020). Several of these strategies have lately gained scientific attention during the COVID-19 pandemic due to the increasing evidence for an involvement of the EG in severely affected patients (Buijsers et al., 2020; Okada et al., 2021; Potje et al., 2021). As with most of the studies investigating the EG, almost all of trials were performed in adults. Furthermore, the treatment effect was mostly monitored by evaluating the EG directly and indirectly, whereas patient outcome was usually not considered.

In summary, the EG is recognized as a critical regulator of vascular integrity and health, and its involvement in acute and chronic diseases affecting the vasculature in adult patients has been well established. In the pediatric population, research concerning the EG is still sparse. Future studies are needed to characterize the normal evolution of the EG during infant and child development, define the contribution of the EG to childhood pathology, evaluate its potential as therapeutic target and prove the benefit of EG preservation/reconstitution on patient outcome.

## Author Contributions

AP-S and CN wrote the manuscript. OG-B revised the manuscript. All authors contributed to the article and approved the submitted version.

## Conflict of Interest

The authors declare that the research was conducted in the absence of any commercial or financial relationships that could be construed as a potential conflict of interest.

## Publisher’s Note

All claims expressed in this article are solely those of the authors and do not necessarily represent those of their affiliated organizations, or those of the publisher, the editors and the reviewers. Any product that may be evaluated in this article, or claim that may be made by its manufacturer, is not guaranteed or endorsed by the publisher.
